# A Parallel Architecture for the Partitioning around Medoids (PAM) Algorithm for Scalable Multi-Core Processor Implementation with Applications in Healthcare

**DOI:** 10.3390/s18124129

**Published:** 2018-11-25

**Authors:** Hassan Mushtaq, Sajid Gul Khawaja, Muhammad Usman Akram, Amanullah Yasin, Muhammad Muzammal, Shehzad Khalid, Shoab Ahmad Khan

**Affiliations:** 1 Department of Electrical & Computer Engineering, Sir Syed CASE Institute of Technology, Islamabad 44000, Pakistan; hassan.mushtaq@ymail.com (H.M.); amanyasin@case.edu.pk (A.Y.); 2 Department of Computer & Software Engineering, CE&ME, National University of Sciences & Technology, Islamabad 44000, Pakistan; sajid.gul@ceme.nust.edu.pk (S.G.K.); shoabak@ceme.nust.edu.pk (S.A.K.); 3 Department of Computer Science, Bahria University, Islamabad 44000, Pakistan; 4 Department of Computer Engineering, Bahria University, Islamabad 44000, Pakistan; shehzad@bahria.edu.pk

**Keywords:** clustering, partitioning around medoids, scalable, parallel, reconfigurable, FPGA, MPSoCs, multi-core processor, time complexity, speedup

## Abstract

Clustering is the most common method for organizing unlabeled data into its natural groups (called clusters), based on similarity (in some sense or another) among data objects. The Partitioning Around Medoids (PAM) algorithm belongs to the partitioning-based methods of clustering widely used for objects categorization, image analysis, bioinformatics and data compression, but due to its high time complexity, the PAM algorithm cannot be used with large datasets or in any embedded or real-time application. In this work, we propose a simple and scalable parallel architecture for the PAM algorithm to reduce its running time. This architecture can easily be implemented either on a multi-core processor system to deal with big data or on a reconfigurable hardware platform, such as FPGA and MPSoCs, which makes it suitable for real-time clustering applications. Our proposed model partitions data equally among multiple processing cores. Each core executes the same sequence of tasks simultaneously on its respective data subset and shares intermediate results with other cores to produce results. Experiments show that the computational complexity of the PAM algorithm is reduced exponentially as we increase the number of cores working in parallel. It is also observed that the speedup graph of our proposed model becomes more linear with the increase in number of data points and as the clusters become more uniform. The results also demonstrate that the proposed architecture produces the same results as the actual PAM algorithm, but with reduced computational complexity.

## 1. Introduction

In recent years, the Internet of Things (IoT) has rapidly grown into one of the most beneficial and dominant communication models for wireless communication applications [1,2,3,4]. Our everyday life is becoming associated with IoT-based entities where the Internet provides a computational platform for data gathered from various sensors. The IoT has hence enhanced the horizon of the Internet making it suitable for different applications. Health care applications in particular have risen in popularity because of rapid development of IoT-based wireless sensor networks, medical devices and wireless technologies [5,6,7].

Healthcare applications mainly deal with large sets of data that need to be classified into various classes. It frequently happens that the output classes are not known a priori, thus unsupervised learning models such as clustering are preferred. Clustering is one of the basic techniques in the data mining domain aimed at organizing a set of data objects into their natural subsets (called clusters), when labels for the data are unavailable. An ideal cluster is an isolated set of data points which are similar to one another, but dissimilar to the points in other clusters [8,9]. Similarity is commonly defined in terms of how close the objects are and is based on a specified distance metric [8]. 

The most fundamental way of clustering is partitioning, which organizes the data into several exclusive groups. More formally, given a set of *N* objects, a partitioning algorithm makes *K* partitions of the data, where each partition represents a cluster. A data point is assigned to a cluster based on the minimum distance measure between the point and the cluster center. The cluster centers are initially chosen either randomly or by using some initialization technique. The algorithm then iteratively improves the formulation of clusters by finding new cluster centers using the objects assigned to the clusters in the previous iteration. All the objects are then reassigned to clusters using updated cluster centers. This process continues until there is no change in the calculated cluster centers in the current iteration compared to the previous one [8]. Clustering has been used in many applications around the globe encompassing many of our real-life applications, including but not limited to healthcare, IoT, academics, search engines, wireless sensor networks, etc. [10,11,12,13,14,15,16,17,18].

Partitioning-based clustering algorithms differ in the way of computing cluster centers, e.g., K-Means [19,20] and K-Modes [21] clustering algorithms use mean and mode values of the clustered data points, respectively, while in K-Medoids [8], clustering method clusters are characterized by their most centrally located objects (called medoids). The first practical realization of the K-Medoids method was introduced as the Partitioning Around Medoids (PAM) algorithm. PAM is more robust than K-Means against noise and outliers, but this robustness comes at the expense of more computations. 

Considering a PAM algorithm which is clustering a dataset with *n* points into *k* clusters, the time complexity required for it to complete its task is roughly $O\left(k{\left(n-k\right)}^{2}\right)$. This makes the algorithm resource and time intensive, especially in the present age where huge amounts of data are at our disposal for processing, thus it becomes a major bottleneck for real time implementation. The utility of the PAM algorithm demands an implementation of the PAM algorithm which is computationally less intensive. This timing complexity of PAM algorithm can be reduced by the introduction of parallel processing and multi-core solutions to the problem. Furthermore, such as design is also suited to today’s state-of-the art multicore and reconfigurable computing hardware platforms such as Graphics Processing Units (GPUs) and Field Programmable Gate Arrays (FPGAs), essentially leading to a computationally inexpensive implementation of the PAM algorithm targeted for real-time processing. The use of FPGAs and reconfigurable computing in faster implementations of various algorithms has gained popularity in recent years. They have been used in various domains ranging from biomedical image/signal processing to data analytics and more [22,23,24]. FPGAs along with SoC designs have also been used extensively in optimizing various algorithms, such as deep learning and HoG-based segmentation, for application development [25,26,27,28,29,30]. 

In this paper we present a scalable parallel architecture of the PAM algorithm which can be implemented either on a multi-core processor system or on a reconfigurable device. The proposed algorithm makes use of divide and conquer approach using multiple processing units. The multiple cores perform homogeneous operations on independent set of data and share results with each other to finalize a single iteration. 

The remainder of the paper is organized as follows: Section 2 provides an overview of the background related to acceleration of the PAM algorithm, Section 3 provides an overview of the PAM algorithm and our proposed architecture of parallel PAM. The results are presented and thoroughly discussed in Section 4, followed by the conclusions of the paper in the last section.

## 2. Background

The complexity of the PAM algorithm has forced researchers to come up with various modifications in order to speed up the algorithm. In this respect a basic multi-core implementation of K-Medoids was proposed which divides the algorithm into sub-tasks, where each sub-task is implemented on a separate core. The proposed design provides improved speedup (4× while utilizing 16 cores) but is limited by the number of hardware cores available at a user’s disposal. A similar multi-core solution has been provided by Rechkalov in [31] for the Intel Xeon Phi Many-Core Coprocessor. The proposed system makes use of OpenMP parallelizing technology and loop vectorization within the algorithm along with the tiling approach. Experimentation showed that the performance of the optimized version of the algorithm is improved but the overall performance depends on the nature of the data to be clustered [31]. Velmurugan et al. discussed the performance improvements that can be made in K-Medoids by simply changing how the data is distributed. In order to prove this concept, normal and uniform distributions of data were used showing that execution time varies as the selected distribution changes [32]. Park et al. proposed an alternate way of computing the K-Medoids algorithm which is similar to the K-Means algorithm [33]. In the proposed solution Euclidean distance is calculated once and then used to calculate new medoids at each iteration. Experiments showed that the execution time of the algorithm was reduced significantly in comparison to the original algorithm.

The increase in popularity of reconfigurable devices, multi-core systems and Graphic Processing Units (GPUs) has seen researchers focusing their efforts on finding parallel models of various machine learning algorithms to improve their performance. A scalable multi-core hardware architecture for implementation of K-Means is proposed in [14] which makes use of tiling to perform multiple tasks in parallel (cores) to speed up the system. The cores are further interconnected using a Network-on-Chip (NoC) interconnect network to offload traffic and provide scalability to the design by minimizing the message passing bottlenecks. The experimentation yielded a near linear speedup with increase in number of cores while the hardware resources and clock speed were not affected. Similarly, a parallel processing model for implementation of the mean shift clustering algorithm for FPGA implementation was proposed by Tehreem et al. in [34]. The proposed model consists of homogenous processing cores running in parallel on independent data subsets. These cores were connected through a bus for data sharing. The proposed model worked in a collaborative working environment where each core works independently and shares its data with others for finalization of results. This model provided a significant speedup while utilizing only 10.31% of the total device slice registers and 33% of total slice LUTs of a Spartan 6 FPGA. A multi-processor architecture having heterogeneous tiles for real time image processing was proposed in [28]. Each tile of the proposed architecture provided computational and memory capabilities. These tiles were connected via a novel NoC structure named Spidergon. The design provided support for different algorithmic classes and ran at 400 MHz ensuring real time processing of up to 30 VGA frames/s [35]. Similar efforts have been made to an optimized hardware design of the Particle Swarm Optimization (PSO) algorithm by Mehmood et al. in [36]. A modified PSO-based technique based on multi-core sequential architecture is presented in the paper. The processing cores implementing the sequential architecture were connected via NoC for implementing a parallel architecture. The architecture was benchmarked against a pure software-based implementation indicating an average speed-up of 22.53 and 29.37 for non-NoC-based HMPSO and a NoC-based MPSO, respectively, over 25 experiments [32]. Li et al. proposed in [37] an efficient error rate, in a proposed VLSI architecture of FCM with Spatial constraints (FCM-S) for image segmentation. To lower the segmentation architecture, the spatial information is used during the FCM training process. In addition, the architecture employs a high throughput pipeline to enhance the computation speed. Experimental results revealed that the proposed architecture implemented on a SoPC architecture attains a speedup of up to 342.51 over its software counterpart. The proposed architecture therefore is an effective alternative for applications requiring real-time image segmentation and analysis.

Existing PAM algorithms and their respective architectures are not that scalable and have an upper bound on the reduction in computational complexity they can achieve. In the proposed system, we present a scalable parallel architecture of PAM algorithm which can exponentially reduce the computational complexity. It introduces the concept of working in a collaborative environment approach by dividing data into multiple processing units which perform homogeneous operations independently and finally give a combined result. 

## 3. Theory and Design of the Parallel PAM Algorithm

### 3.1. Overview of the PAM Algorithm

We will use the following notation to formally describe the PAM algorithm. Let *X* = {*x*_1_, *x*_2_, *x*_3_, …, *x_N_*} be the set of *N* data points to be clustered where each data point consists of *d* real-valued attributes. Let *M* = {*m*_1_, *m*_2_, *m*_3_, …, *m_K_*} is a set of *K* medoids such that *M* *⊂ X*, where *K* is the number of clusters such that *K << N*. *D: X* × *M → R* is a distance metric, usually it is a matrix of Euclidean distances from each object *x_i_* to its nearest medoid *m_j_*. In each iteration of algorithm, a pair of medoid object *m_j_* and non-medoid object *x_i_* is selected which produces the best clustering when their roles are swapped. The objective function used is the sum of the distances from each object to its closest medoid:

(1) $$Cost=\overset{N}{\underset{i=1}{\sum }}\underset{1\;\leq j\;\leq K}{min}D\left(x_{i}\;,\;m_{j}\right)$$

The algorithm PAM proceeds in the following manner:

- In the first phase (called Build Phase) an initial clustering is obtained by the successive selection of *K* medoids. The first medoid is the one for which the sum of distances to all non-medoid objects is minimum. This is actually the most centrally located data point in set *X*. Subsequently, at each step another object is selected as a medoid, for which the objective function is minimum. The process is continued until *K* medoids have been found.
- In the second phase of the algorithm (called Swap Phase), it is attempted to improve the set *M* of medoids and therefore the clustering obtained by this set. The algorithm goes through each pair of objects (*m_j_*, *x_h_*), where *m_j_* is a medoid and *x_h_* is non-medoid object and *x_h_* belongs to cluster *j*. The effect on the objective function is determined when a swap is carried out i.e., when object *x_h_* is considered as a medoid in place of object *m_j_*. For each cluster *j*, the object *x_h_* is selected as its new medoid for which the objective function is minimized and thus the set *M* is updated. This process is iterated until no further decrease in objective function value is possible or in other words there is no update in set *M* between two consecutive iterations.

### 3.2. Proposed Design Flow

The aim of this paper is to propose a model to make PAM algorithm computationally less expensive by parallelizing its functionality such that it uses less resources when implemented on reconfigurable hardware. The whole working of our research revolves around the concept that how well we parallelize the PAM algorithm so that its overall computational complexity can be significantly reduced. We concluded that this task can be performed well by following these steps:

1. Dividing the algorithm into a well-defined sequence of subtasks.
2. Identifying the portions of the algorithm which can be executed in parallel.
3. Running these subtasks for equal subsets of data simultaneously on multiple homogeneous cores.
4. Combining the intermediate results from different PEs to produce a final clustering.

#### 3.2.1. Sub-Tasking of PAM Algorithm

**Figure 1:**
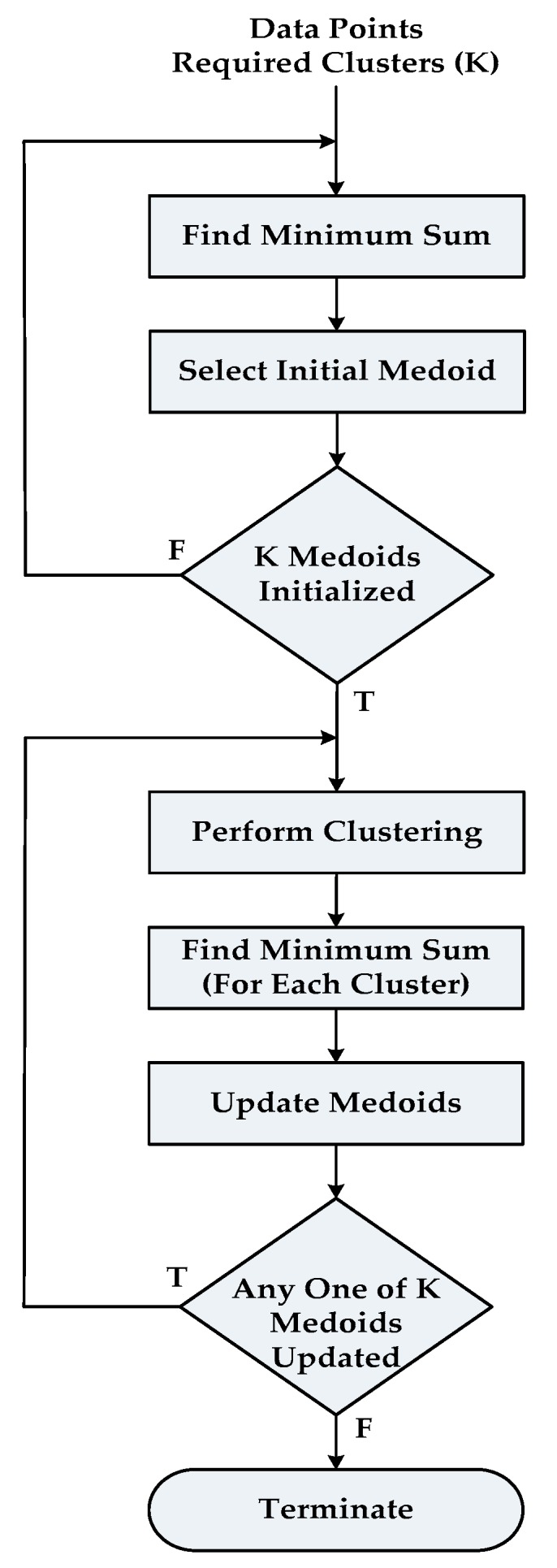
Flow chart of the sequential PAM algorithm.

As we discussed in the previous section, the PAM consists of two phases called (1) Build Phase and (2) Swap Phase. The complete flow chart of the Partitioning Around Medoids algorithm in terms of this is shown in Figure 1.

The peudo-code of PAM is given in Algorithm 1 below.

**Algorithm 1.** Pseudo-code of PAM**Procedure:** Partitioning Around Medoids (PAM)**Input:***K* (No. of clusters), *X* (Data Set)**Output:***C* (Vector containing cluster tags against each data object), *M* (Medoids)1. Initialize *M*        /* Build Phase */2. **repeat**        /* Swap Phase */3.  Find clusters4.  Perform swapping and update Medoids5. **until** no update in any of *K* Medoids

We start by splitting the working Build Phase into two subtasks namely: (1) Find Minimum Sum and (2) Select Initial Medoid. In the first subtask, one by one each object is temporarily selected as a medoid and the minimum value of the objective function is computed for the set of medoids selected up to current step. The second subtask selects the temporary medoid as the actual medoid for which the objective function value is minimum. Algorithm 2 depicts the pseudo-code of the Build Phase.

Then the Swap Phase is split into three subtasks called: (1) Perform Clustering, (2) Find Minimum Sum (For Each Cluster) and (3) Update Medoid. The first Subtask assigns each object to its closest medoid to form clusters. In the second subtask, one by one the role of each object within a cluster is swapped with its medoid and smallest value of the cost function is computed.

**Algorithm 2.** Pseudo-code of the Build Phase1. **repeat**2.  **for** a := 1 → N **do**        /* Find Minimum Sum */3.   Select X_a_ as temporary Medoid4.   **for** i := 1 → N **do**5.    **for** each Medoid selected yet (including X_a_) **do**6.     Find the minimum Euclidean distance b/w X_i_ and Medoid7.    **endfor**8.    Find sum of minimum distances9.   **endfor**10.    Find minimum sum11.  **endfor**12.  Select X_a_ as actual Medoid for which the sum is minimum    /* Select Initial Medoid */13. **until** ‘K’ initial Medoids are selected

The last subtask of this phase updates the medoid of the current cluster for which the cost function value is minimum. Subtasks 2 and 3 are repeated for all clusters. The corresponding pseudo-code is shown as Algorithm 3.

**Algorithm 3.** Pseudo-code of the Swap Phase1. **repeat**2.  **for** i := 1 → N **do**      /* Perform Clustering */3.          **for** j := 1 → K **do**4.            Find Euclidean distance b/w X_i_ and M_j_5.        Tag X_i_ with j for which this distance is minimum6.      **endfor**7.  **endfor**8.  **for** j := 1 → K **do**      /* Find Minimum Sum (For Each Cluster)*/9.     **for** each data point X_a_ ϵ cluster j **do**10.       **for** each data point X_i_ ϵ cluster j **do**11.           Find sum of Euclidean distances b/w X_a_ and X_i_12.       **endfor**13.       Find minimum sum14.      **endfor**15.      Update X_a_ as j^th^ Medoid for which the sum is minimum    /* Update Medoid*/16.  **endfor**17. **until** no update in any of ‘K’ Medoids

Now in order to reduce the computational complexity and improve the execution time, the PAM algorithm needs parallelism. We have identified from Algorithm 2 that the subtask 1 of Build Phase can be executed in parallel on multiple PEs for equal subsets of data, while subtask 2 will compute the final result of this phase. Similarly, it is clear from Algorithm 3 that subtasks 1 and 2 of the Swap Phase can be parallelized well.

#### 3.2.2. Paralleling the PAM Algorithm and Proposed Architecture

**Figure 2:**
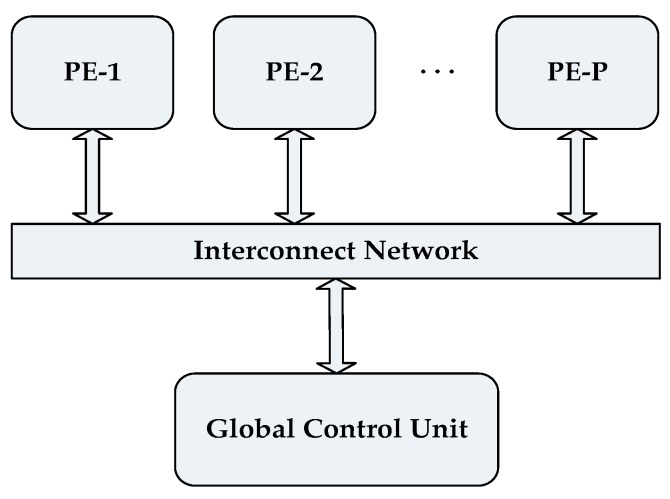
Top level block diagram of the proposed architecture.

Our proposed architecture uses *P* number of homogeneous cores or Processing Elements (PEs) which are connected through an interconnect network such as a bus as shown in Figure 2. Each PE has given access to all data points thus it can work in parallel with other PEs to achieve faster convergence and eventually an increased throughput. A Global Control Unit is used to control the overall flow of the algorithm. The interconnecting network used in the design can be a bus-based, point to point or network-on-chip-based interface. The choice of interconnection is based on the complexity and requirement of the applications, e.g., a NoC based interface will provide better concurrent message passing at the cost of area and power overhead.

The overall working of the parallel PAM for this multi-core processor model is described in the following steps:

1. A data set of size *N* is made completely divisible into the number of available cores *P* by appending zeros at the end of the data set so that equal subsets can be assigned to each core.
2. The complete data set *X* is replicated in all available PEs and equal partitions of *X* are assigned to each PE.
3. Each PE then executes the subtask “Find Minimum Sum” of the Build Phase for its respective data subset of size $\frac{N}{P}$ in parallel. Master PE (any processing element can be assigned to perform as master PE because all PEs are homogenous) will collect the results of the first subtask from each PE and perform the subtask “Select Initial Medoid”. This step is repeated until *K* medoids are initialized, as described in Algorithm 4 below.
4. Final results of Build Phase are sent to all PEs so that they can proceed to the next phase of algorithm.
5. Each PE will tag all its assigned data objects with their closest cluster numbers. These tags are stored in local memory associated with each data object. All PEs one by one broadcast their $\frac{N}{P}$ tags over the interconnect network so that each PE can have complete result of clustering.
6. The subtask “Find Minimum Sum (For Each Cluster)” of the Swap Phase is executed by each core in parallel. A master PE will perform the subtask “Update Medoid” after receiving results from other PEs. Steps 5 and 6 are repeated until no update in any of *K* medoids is reported. Algorithm 5 depicts the working of the Swap Phase in case of parallel PAM.

**Algorithm 4.** Pseudo-code of the Build Phase for Parallel PAM1. **repeat**2.  **for** p := 1 → P **do in parallel**3.   **for** each data point X_a_ ϵ p **do**       /* All PEs do in parallel*/4.    Select X_a_ as temporary Medoid5.    **for** i := 1 → N **do**6.     **for** each Medoid selected yet (including X_a_)**do**7.      Find the minimum Euclidean distance b/w X_i_ and Medoid8.     **endfor**9.     Find sum of minimum distances10.    **endfor**11.    Find minimum sum12.   **endfor**13.  **endfor**14.  **for** p := 1 → P **do**        /* Only master PE will do this */15.   Select X_a_ as actual Medoid for which the sum is minimum from all PEs16.  **endfor**17. **until** ‘K’ initial Medoids are selected

**Algorithm 5.** Pseudo-code of the Swap Phase for Parallel PAM1. **repeat**2.  **for** p := 1 → P **do in parallel**3.   **for** each data point X_i_ ϵ p **do**      /* All PEs do in parallel*/4.    **for** j := 1 → K **do**5.     Find Euclidean distance b/w X_i_ and M_j_6.     Tag X_i_ with j for which this distance is minimum7.    **endfor**8.   **endfor**9.   **endfor**10.  Concatenate clustering results from all PEs      /* All PEs do this*/11.  **for** p := 1 → P **do in parallel**12.   **for** j := 1 → K **do**        /* All PEs do in parallel*/13.    **for** each data point X_a_ ϵ p & cluster j **do**14.     **for** each data point X_i_ ϵ cluster j **do**15.      Find sum of Euclidean distances b/w X_a_ and X_i_16.     **endfor**17.     Find minimum sum for each cluster18.    **endfor**19.   **endfor**20.  **endfor**21.  **for** j := 1 → K **do**        /* Only master PE will do this */22.   **for** p := 1 → P **do**23.    Update X_a_ as j^th^ Medoid for which the sum is minimum from all PEs24.   **endfor**25.  **endfor**26. **until** no update in any of ‘K’ Medoids

**Figure 3:**
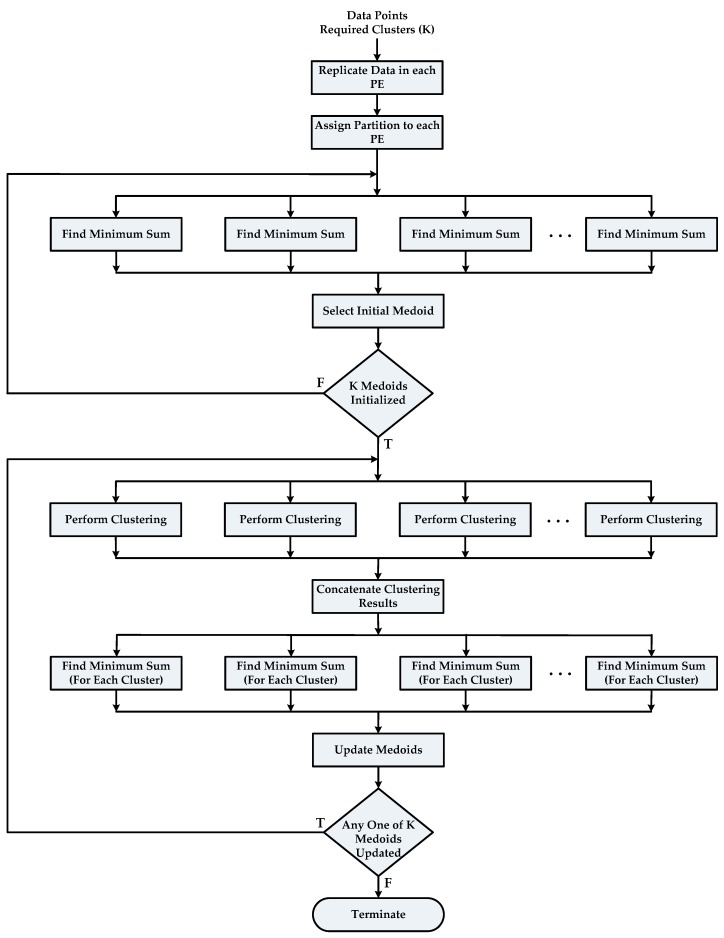
Flow chart of the parallel PAM algorithm.

The complete work flow of the parallel PAM algorithm is depicted in Figure 3 below.

**Figure 4:**
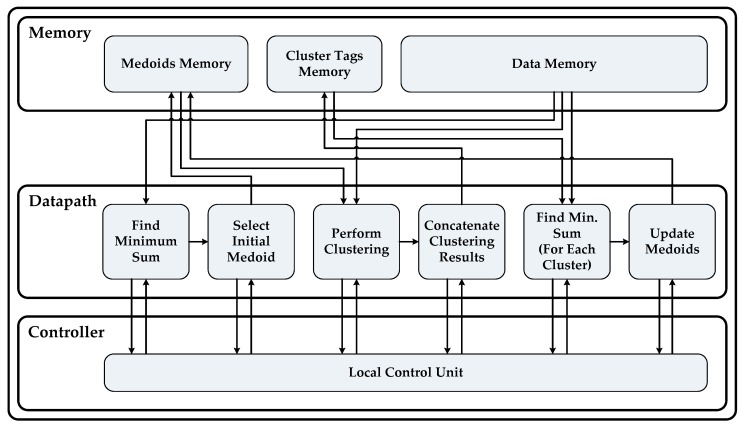
Internal structure of a processing element (PE).

At this stage we can explore the internal structure of a processing element at an abstract level. As shown in Figure 4, each PE is composed of three sections (1) Controller, (2) Datapath and (3) Memory. The Controller section consists of a local control unit to manage the overall sequencing of subtasks within a processing element and to manage communication with other PEs. The Datapath section contains sub-blocks which are basically hardware sub-modules implementing the functionality of different sub-tasks of the algorithm. Finally, each core has a memory section which consists of a memory block of size *N* × *d* to hold the complete data set, a memory block of size *M* × *d* to store final values of medoids and an *N* × 1 sized block of memory to store cluster tags against each data object.

## 4. Experimentation and Results

In order to demonstrate the usefulness of our proposed parallel implementation of PAM, first we implemented the sequential PAM algorithm as described in the previous section and recorded the running time of the algorithm in terms of number of computations required by both the build and swap phases. Then the running time in the case of parallel implementation of PAM was computed along similar lines to examine the speedup attained for different numbers of PEs. In the latter case, the time required for communication among different PEs, to share results and data, was also added to get the total running time. 

First, this experimentation was performed for randomly generated artificial data points (*N* = 400 to 1600) each having two attributes (*d* = *2*). At the second stage, color-based segmentation of different RGB images was performed. Sizes were around 7000 pixels to 16,000 pixels for different images and value of *d* is 3 in this case (three color components). After thorough experimentation, the following results were concluded.

**Figure 5:**
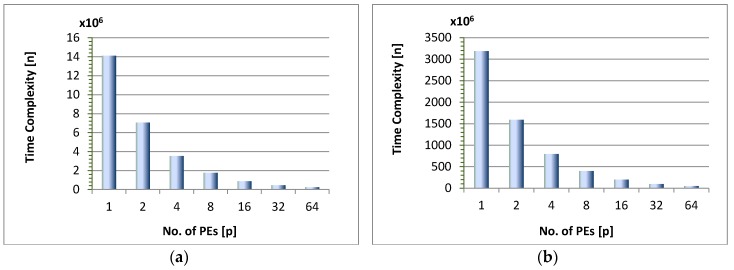
Computational complexity of the proposed architecture: (**a**) For artificial random data, *N* = 800, *d* = 2 & *K* = 4; (**b**) For image pixels, *N* = 9720, *d* = 3 & *K* = 4.

**Figure 6:**
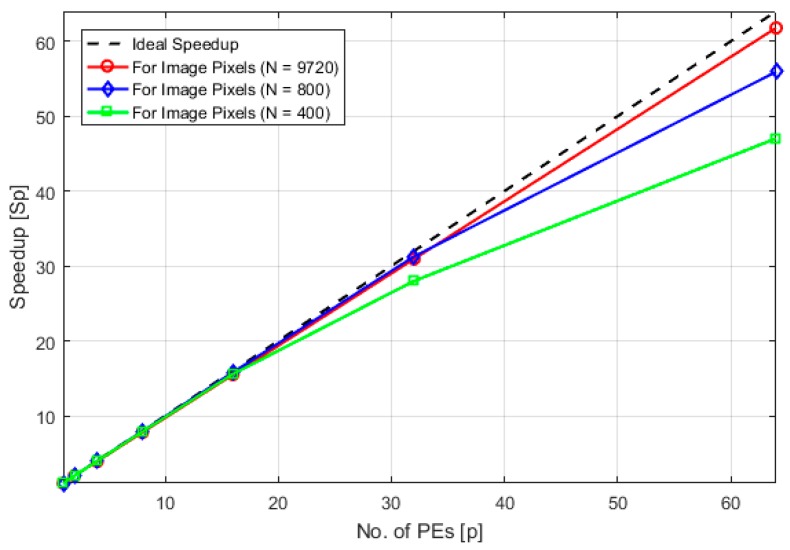
Comparison of speedup for different dataset sizes.

1. The time complexity *n* of the algorithm reduces exponentially as we increase the number of cores for the same data set or image. Here by time complexity we mean the running time which is taken by all computations required by the build and swap phases. This computation complexity reduces as we increase the number of processing entities and divide the computations among them. For example, the running time of PAM algorithm for *N* = 800, *d* = 2, *K* = 4 and *P* = 1, is *n* ≈ 1.41 × 10^7^. For *P* = 2, this value is half of the previous value i.e., *n* ≈ 7.1 × 10^6^ plus a small communication overhead = 4948. Similarly, for *P* = 4, *n* ≈ 3.5 × 10^6^ plus overhead is 3416 and so on. Figure 5 shows this trend for both the artificial data set of size 800 and an image of 9720 pixels in size. Furthermore, it is observed that when the size of the data is increased this trend becomes more uniform and gets close to $\frac{n_{1}}{P}$, where *n*_1_ is the computational complexity of the sequential algorithm. This is evident from Figure 5a,b where the trend remains the same even if we increase the data points which need to be clustered.
2. The speedup of the algorithm is defined as *S_p_* = $\frac{n_{1}}{n_{p}}$, where *n*_1_ is running time of the algorithm for a single PE and *n_p_* is the running time for *P* processing elements. It was observed that the speedup of the parallel PAM algorithm increases with the increase in the number of processing elements, but this increasing trend varies slightly for different scenarios discussed below.
  (1) The speedup graph gets more linear as the data size increases for the same number of PEs. This trend is shown in Figure 6.
  (2) If the clusters to be formed are uniform i.e., the number of data objects is equal in each cluster then the speedup attained is slightly better than the case when clusters are non-uniform for same data set size.

As we know PAM consists of two discrete phases, the first one is the Build Phase which is just an initialization method for medoids while the actual algorithm which iteratively runs and tries to minimize the objective function is the Swap Phase. The Build Phase is computationally more expensive than the Swap Phase but on the other hand it significantly increases the probability of convergence of the PAM algorithm. Other different methods of initialization can also be used, the simplest one of which would be random initialization of medoids, but at the cost of a decrease in the probability of convergence.

**Figure 7:**
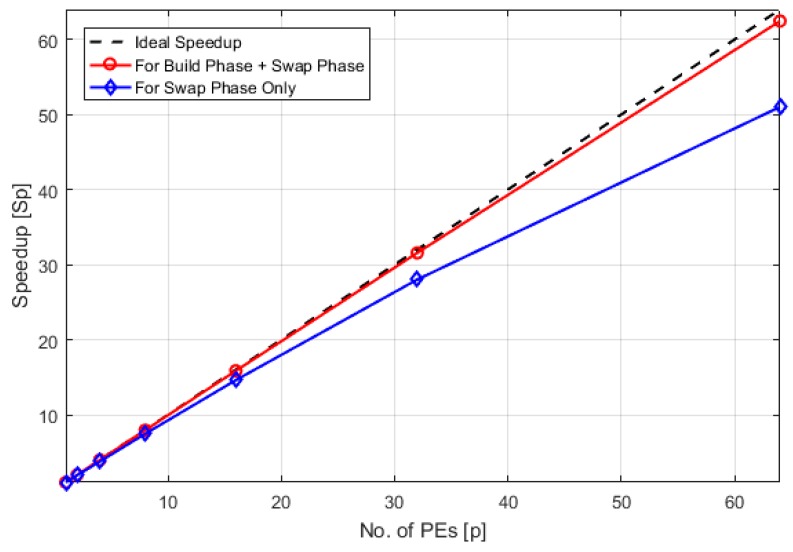
Comparison of speedup for the case when medoids are initialized using the build phase with the case when medoids are randomly initialized, data size is same in both cases.

(1) It was observed that due to the high computation cost of the Build Phase, the total computation cost of the algorithm (order of *N*^2^) is much higher than the communication cost (order of *N*), for large values of *N*. Therefore, communication overhead doesn’t affect the speedup and it is almost equal to the number of PEs.
(2) If we don’t include the effect of build phase or medoids are randomly initialized then communication overhead will affect the speedup achieved by our parallel PAM algorithm, otherwise this trend will be near linear, as shown in Figure 7.

**Figure 8:**
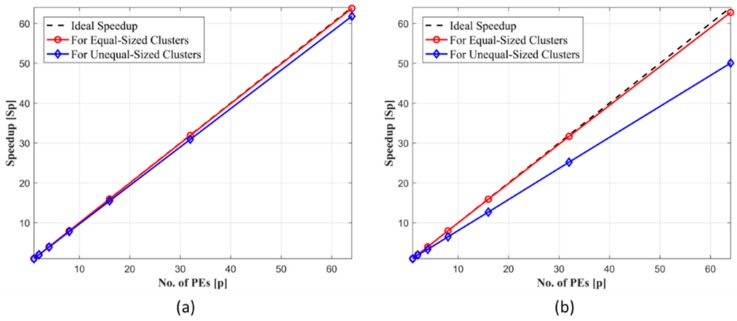
(**a**) Comparison of speedup for same size of data but having equal and unequal cluster sizes (**b**) Comparison of speedup for equal and unequal sized clusters but data size is same for both cases. Effect of Build Phase is not included.

We have also studied the effect of cluster size on speedup. If the clusters to be formed have equal sizes, i.e., the number of data objects is equal in each cluster then the speedup graph is slightly better than the case when all clusters have different sizes. This effect is shown in Figure 8a for clustering of an RGB image of size *N* = 9720 pixels when total number of computations including both Build Phase and Swap Phase is taken into account. When the effect of only Swap Phase is considered then there is a prominent difference between speedup achieved by the algorithm for equal-sized and unequal-sized clusters as opposed to the previous case when the effect of both phases is considered. These results are shown in Figure 8b.

**Figure 9:**
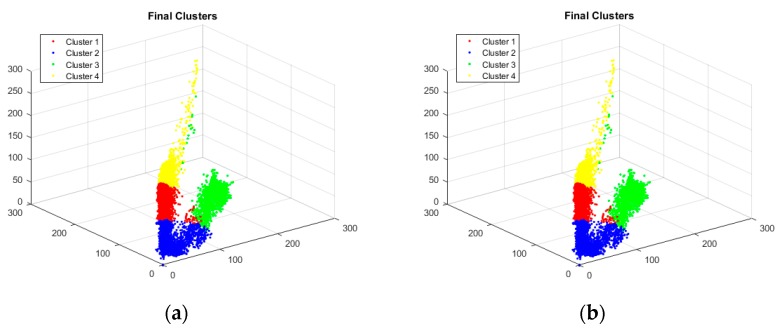
Clusters produced by (**a**) our proposed implementation of parallel PAM with *P* = 64 and (**b**) K-Medoids clustering algorithm [17]. Final medoids locations and objective function value were same in both cases.

Finally, the accuracy of both sequential and parallel implementations of PAM was checked against the results obtained through a well-known but different implementation of the K-Medoids clustering algorithm [17]. This implementation is being used by many researchers in the field of cluster analysis. It was found that the results, such as cost function value, medoids values and cluster tags against each data point, obtained by our implementations and the referenced algorithm for same data input were exactly matched. We have performed color-based segmentation of different images to verify the accuracy of our proposed model. Figure 9 shows a comparison of the final clustering of pixels of an example image in 3-dimensional space.

**Figure 10:**
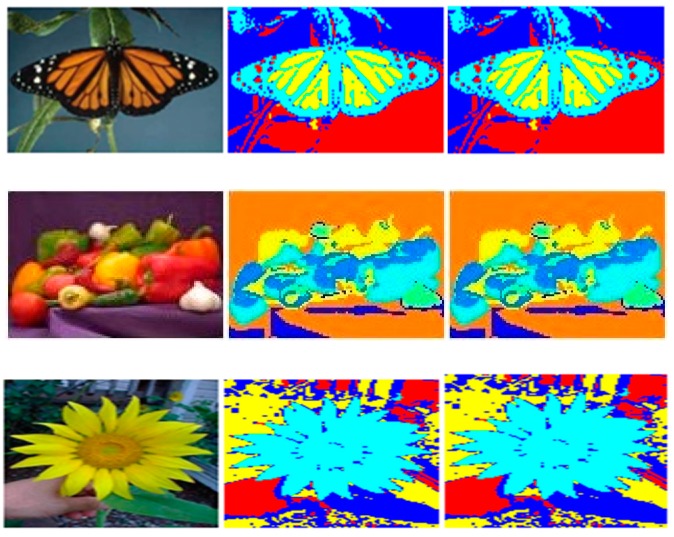
Left to right: original image, result of proposed implementation of PAM, result of K-Medoids clustering algorithm [17] (SSIM = 1, for all cases).

**Figure 11:**
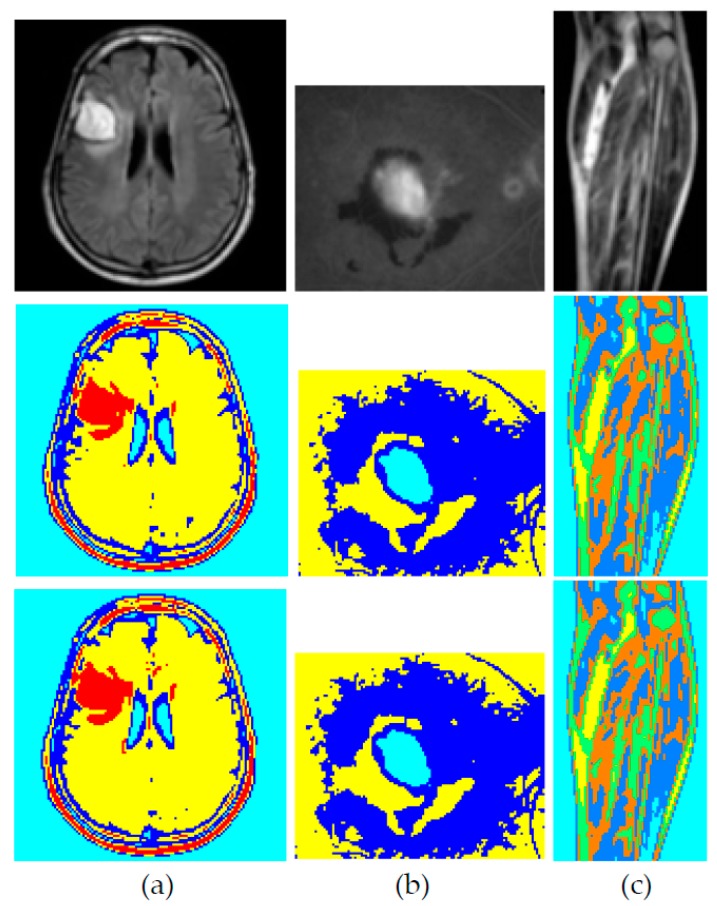
Top to bottom: original image, segmentation result of proposed model of PAM algorithm, result of K-Medoids clustering by [17]: (**a**) MRI scan of a brain to detect tumor (T2W image with full brain coverage in axial plane), SSIM = 0.97; (**b**) Fluorescein angiography of a retina to detect neovascular AMD, SSIM = 1.00; (**c**) MRI scan of a leg to detect fluid collection in calf muscles due to injury (T2W image in coronal plane), SSIM = 0.93.

In order to assess the quality of the output images, the Structural Similarity Index Metric (SSIM) has been calculated. The Structural Similarity Index (SSIM) is an assessment mechanisms which computes the image quality degradation which is caused by processing images [38]. SSIM has been calculated for the output images of this segmentation process and was found to be 1.00 for all images. Some example images and output images are shown in Figure 10. Similarly, SSIM of the proposed architecture was cross-checked on segmentation of various health-related images such as MRI scans of brain tumor, fluorescein angiography of a retina to detect neovascular AMD and MRI scans of legs for detection of fluid collection in calf muscles due to injury which resulted in values of 0.97, 1.0 and 0.93, respectively, as shown in Figure 11. The results indicate that the proposed optimized model can be applied on images for segmentation processes without fear of distortion of the results as is clear from SSIM.

In this article, we have designed a generic scalable architecture for K-Medoids clustering which has been tested for randomly generated data sets and color image segmentation. The proposed model is scalable with respect to the number of processing cores depending on the availability of hardware resources. We can increase the parallelism by increasing the number of PEs. The same architecture is also valid for any number of clusters and also for any dimensional data points. The results presented in Figure 6 and Figure 7 use the Euclidean distance metric to find similarity (or dissimilarity) among data objects but this architecture is also tested for other distance metrics such as squared Euclidean, city block etc. it was found that speedup trend is not affected by using different distance metrics.

The proposed architecture is an ideal fit for implementing it on reconfigurable devices such as FPGAs and MPSoCs because of its overall small footprint. Each sub-task is designed in such a way that its internal working can easily be unfolded, while keeping the hardware resources in check, to further reduce the overall processing time of algorithm. The efficiency of the proposed at higher number of PE shows divergence from ideal speedup, this trend can be improved by the use of efficient communication interface such as Network-on-Chip (NoC) for linking of multiple cores. Researchers have provided various platforms for NoC based MPSoC models [39,40]. The use of NoC in multicore models in various application has shown promising results in terms of scalability and efficiency [14,41,42,43,44,45].

## 5. Conclusions

Clustering is a commonly used platform for labeling of unknown data based on similarity. Among the many clustering scheme variants the Partitioning Around Medoids (PAM) algorithm belongs to the partitioning-based methods of clustering used for numerous applications ranging from object categorization to data compression. In today’s world a significant increase in available data for clustering has made clustering algorithms computationally expensive thus they can’t be used in their original form for real-time processing. In this paper a scalable multi-core parallel architecture for implementation of PAM is presented. The architecture is aimed towards dividing the algorithm into sub-tasks and implementing them in parallel in order to reduce the computation time. The proposed architecture has been designed while keeping in view the reconfigurable architectures such as FPGA and MPSoC or for multi-core processor platform. Our model equally divides the data among available processing cores which perform homogeneous tasks in parallel on respective local data points. The intermediate results of each core are shared with other cores for finalizing the ultimate clusters thus forming a collaborative working environment (CWE). Experiments were carried out on randomly generated datasets and colored images. The computational time of our proposed solution was compared against sequential implementation of the PAM algorithm. The results showed an exponential decrease in the computational complexity of the algorithm with the increase in the number of processing cores. Similarly, the speedup trends showed an almost linear increase against the sequential algorithm. It was also observed that speedup of the proposed implementation becomes more linear as the size of the data set increases and also as the clusters become more uniform for the same data set size. In the future, the proposed algorithm can be implemented using an effective communication interface such as NoC in order to reduce the communication overhead causing non-linearity of speedup which occurs at a high number of cores. Furthermore, more experimentation can be done on real-time video feeds to validate the effectiveness of the algorithm and increase its application base to include video processing as well.
